# Integrating Google Trends Search Engine Query Data Into Adult Emergency Department Volume Forecasting: Infodemiology Study

**DOI:** 10.2196/32386

**Published:** 2022-04-22

**Authors:** Jesus Trevino, Sanjeev Malik, Michael Schmidt

**Affiliations:** 1 Department of Emergency Medicine The George Washington University School of Medicine & Health Sciences Washington, DC United States; 2 Department of Emergency Medicine Northwestern University Feinberg School of Medicine Chicago, IL United States

**Keywords:** infodemiology, patient volume forecasting, emergency medicine, digital health, Google Trends, infoveillance, social media, prediction models, emergency department

## Abstract

**Background:**

The search for health information from web-based resources raises opportunities to inform the service operations of health care systems. Google Trends search query data have been used to study public health topics, such as seasonal influenza, suicide, and prescription drug abuse; however, there is a paucity of literature using Google Trends data to improve emergency department patient-volume forecasting.

**Objective:**

We assessed the ability of Google Trends search query data to improve the performance of adult emergency department daily volume prediction models.

**Methods:**

Google Trends search query data related to chief complaints and health care facilities were collected from Chicago, Illinois (July 2015 to June 2017). We calculated correlations between Google Trends search query data and emergency department daily patient volumes from a tertiary care adult hospital in Chicago. A baseline multiple linear regression model of emergency department daily volume with traditional predictors was augmented with Google Trends search query data; model performance was measured using mean absolute error and mean absolute percentage error.

**Results:**

There were substantial correlations between emergency department daily volume and Google Trends “hospital” (*r*=0.54), combined terms (*r*=0.50), and “Northwestern Memorial Hospital” (*r*=0.34) search query data. The final Google Trends data–augmented model included the predictors Combined 3-day moving average and Hospital 3-day moving average and performed better (mean absolute percentage error 6.42%) than the final baseline model (mean absolute percentage error 6.67%)—an improvement of 3.1%.

**Conclusions:**

The incorporation of Google Trends search query data into an adult tertiary care hospital emergency department daily volume prediction model modestly improved model performance. Further development of advanced models with comprehensive search query terms and complementary data sources may improve prediction performance and could be an avenue for further research.

## Introduction

### Background

Internet-based technologies and web-based services have facilitated new ways of seeking and communicating health-related information. A valuable aspect of web-based information transactions is the record of communication itself, which, in aggregate, may reflect population-level behaviors. For example, researchers have used search engine queries and volumes, such as Google Trends, to attempt to recognize population behavior–based patterns. Examples of this research are found in many industries, such as finance [1] and criminology [2].

The emerging field of infodemiology is defined by Eysenbach [3] as “the science of distribution and determinants of information in an electronic medium, specifically the Internet, or in a population, with the ultimate aim to inform public health and public policy.” The major debut application of infodemiology within the health care industry involved monitoring the seasonal emergence and peak of influenza with Google Flu Trends [4], which initially outperformed the extant gold standard FluNet from the Centers for Disease Control and Prevention; however, Google Flu Trends later suffered from poor predictions attributed to model overfitting, among other reasons [5].

The field of infodemiology has grown substantially in the past decade, in terms of disease applications and data sources. In early infodemiology research, the majority of papers involved the study of influenza; more recent reviews [6,7] detail an expanded scope of subject matter, such as influenza, multiple sclerosis, suicide, prescription drug abuse, and e-cigarettes, and the most common data sources included Twitter (45%), Google (24.6%), other websites (13.9%), blogs (10.1%), and Facebook (8.9%). In addition to research applications, one review [8] described the following practical applications of infodemiology by health care organizations: infoveillance, dissemination of health information, misinformation management, and health interventions. Most recently, during the COVID-19 pandemic, researchers have used infodemiology to study public opinion toward COVID-19 vaccines [9] and public health containment measures [10], capture the most frequently asked questions regarding COVID-19 vaccines [11], augment the performance of conventional prediction models for COVID-19 infections [12], and characterize the partisan differences of US legislators in the initial phase of this pandemic [13].

### Prior Work

In infodemiology, data reflecting the use of the internet in seeking health information have been used to improve emergency department patient volume predictions and optimize emergency department resource allocation [14-16]. A Swedish study [14] of emergency department patient volume found that the use of a popular public health website’s traffic volume as a predictor yielded an impressive mean absolute percentage error (MAPE) of 4.8%, which demonstrated that web-based information seeking behaviors can be a useful leading indicator of acute care encounters [14]. A study in the United States found that 86% of participants, who had been recruited from an emergency department waiting room, utilized Google search in the week prior to their emergency department visit; 15% of their searches had been health-related and two-thirds of these searches had been either related to their current chief complaint or for information related to the emergency department and hospital [15]. In addition, internet health information–seeking behavior has been described as a method for patients to prepare questions for upcoming medical appointments with health care providers [16].

Prior studies have used Google Trends search query data to forecast influenza-like illness cases [17] and pediatric daily volumes [18]; however, no studies have evaluated the ability of Google Trends search query data related to chief complaints and health care facilities to predict the overall daily volume in an adult emergency department.

### Study Goal

The ability to predict deviations in typical weekly patterns of emergency department patient volumes could provide emergency department administrators with a valuable tool to optimize resource allocation. We explored the use of Google Trends search query data of chief complaints and health care facilities to improve the prediction performance of adult emergency department daily patient volume.

## Methods

### Emergency Department Encounter Data

Emergency department daily patient volume data were collected from Northwestern Memorial Hospital, a tertiary care adult center located in Chicago, Illinois with an annual volume of 88,000 patient encounters. Data were collected retrospectively from the institution’s databases and included 159,769 emergency department patient encounters that occurred in the period from July 1, 2015 to June 30, 2017. These data included patient arrival date and time, and Emergency Severity Index (levels 1 through 5 in decreasing order of case urgency) [19]. For analysis, data were aggregated by date and Emergency Severity Index.

### Environmental Data

To develop prediction models to be used as a point of reference, we used calendar day (ie, day of week, month) and weather-related variables to derive a traditional emergency department forecasting model. Daily weather data were obtained from the National Centers for Environmental Information and included average temperature, maximum temperature, minimum temperature, precipitation (categorical), and snow (categorical) [20].

### Google Trends Data

#### Data Collection

Google Trends search query data were accessed from the Google Trends API service on June 19, 2018 [21].

#### Keyword Selection

Based on clinical experience and expert opinion, we generated a list of Google Trends terms that would be relevant to an individual seeking health information (ie, terms that would be part of their search engine query) prior to a health care encounter. The terms, which included “emergency department,” “Northwestern Memorial Hospital,” “hospital,” “WebMD,” “chest pain,” “back pain,” “abdominal pain,” “stomach pain,” “side pain,” “fever,” “cough,” “shortness of breath,” “headache,” “numbness,” “weakness,” “blood urine,” and “blood stool,” corresponded to 3 broad categories: health care facility, reputable website, and general chief complaints encountered in the emergency department.

#### Region and Period Selection

Google Trends search query data were limited to the Chicago metropolitan area by constraining the API request to the Chicago Nielsen Designated Market Area (code 602) and to daily relative search frequencies from July 1, 2015 to June 30, 2017.

#### Feature Engineering

To engineer a feature that reflected a more precise region around the study hospital, we derived an independent variable: the search query ratio of “Northwestern Memorial Hospital” over “hospital.” We also created a combined variable, which aggregated all Google Trends search query data into a single measure. We performed the following transformations on Google Trends search query variables to explore temporal associations and to engineer features that smooth out short-term fluctuations: 1-day lag, 1-day percentage change, 3-day moving average, and 7-day moving average. After these transformations, a total of 85 Google Trends search query terms were included in the candidate set of predictor variables. Given the difference in scales, Google Trends search query data were standardized before their inclusion as predictor variables in the regression analysis.

### Exploratory and Correlational Analysis

We performed visual analysis of Google Trends search query data and calculated Pearson correlation coefficients between emergency department daily volume and Google Trends variables.

### Model Development and Evaluation

We utilized multiple linear regression, one of the most common methods for emergency department patient volume forecasting and for predictive modeling with Google Trends search query data [22], to create separate predictive models for overall emergency department patient volume and for patient volume by Emergency Severity Index (ie, 1 through 5).

We also created a baseline model with traditional variables, such as calendar day and weather, similar to prior literature [23]. Predictor variable selection was performed using recursive feature elimination, which is a type of backward selection algorithm that offers a systematic approach to variable selection by constructing multiple models with permutations of predictor variables and selecting a parsimonious model that optimizes a prediction performance metric [24]. To evaluate the ability of Google Trends search query data to improve forecasting performance, we augmented the baseline model with Google Trends variables and used recursive feature elimination to identify the highest impact predictor variables.

Models were trained using 10-fold cross-validation, and model performance was assessed using mean absolute error (MAE) and MAPE of prediction values in relation to actual values. Analysis was conducted using R software (version 4.1.0; The R Project) and utilized the caret package (version 6.0-88; Max Kuhn) [25].

### Ethics

This study was considered exempt from review by the Northwestern Memorial Hospital Institutional Review Board because emergency department data were deidentified and contained no protected health information.

## Results

### Exploratory Analysis

The median total emergency department daily volume over this period was 242 patients per day (range 152-305 patients per day; Emergency Severity Index 1: 4043/159,769, 2.5%; Emergency Severity Index 2: 63,611/159,769, 39.8%; Emergency Severity Index 3: 64,091/159,769, 40.1%; Emergency Severity Index 4: 23,773/159,769, 15.0%; Emergency Severity Index 5: 2300/159,769, 1.4%; Emergency Severity Index not available: 1951/159,769, 1.2%).

**Figure 1:**
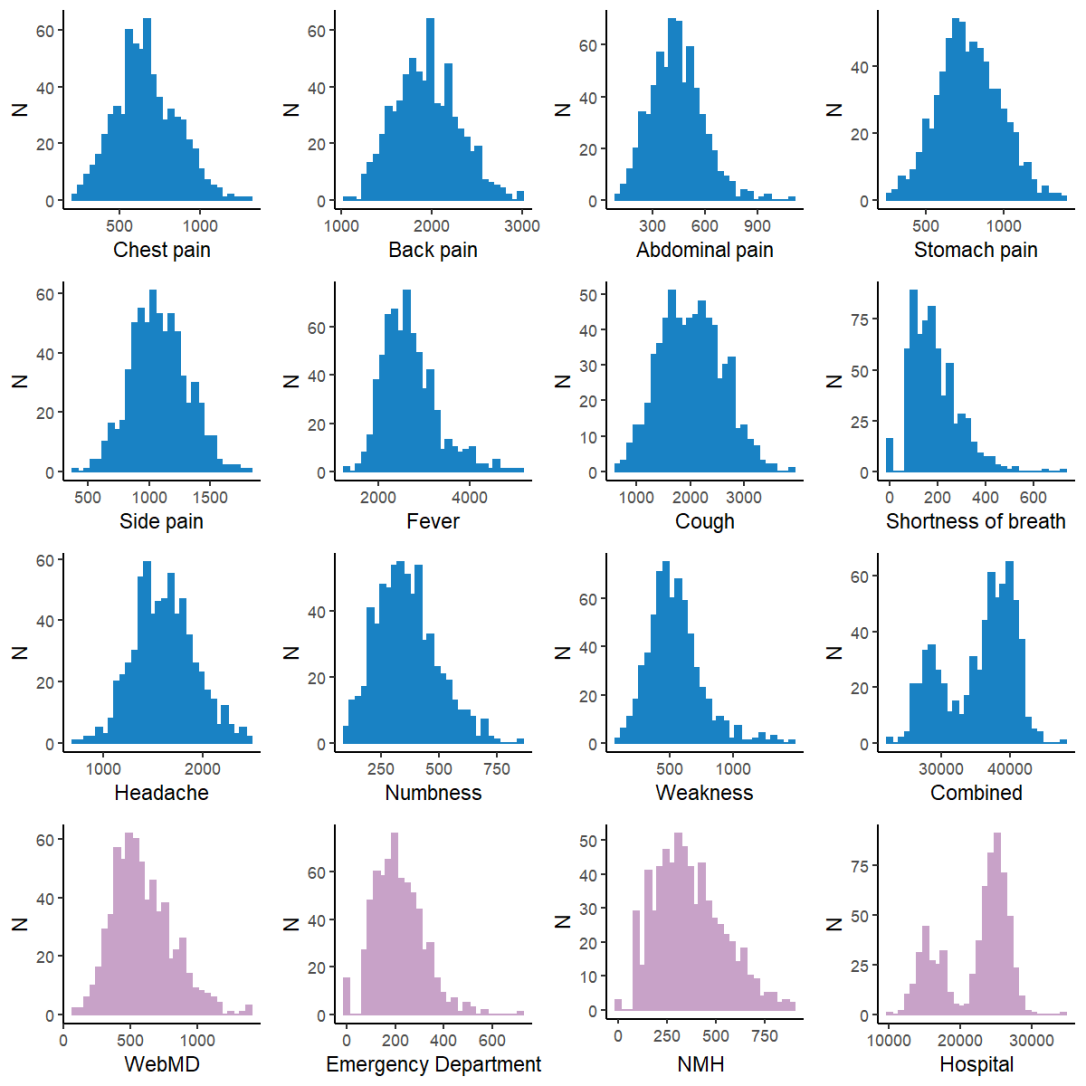
Histograms of candidate Google Trends search query data. N: count; NMH: Northwestern Memorial Hospital.

**Figure 2:**
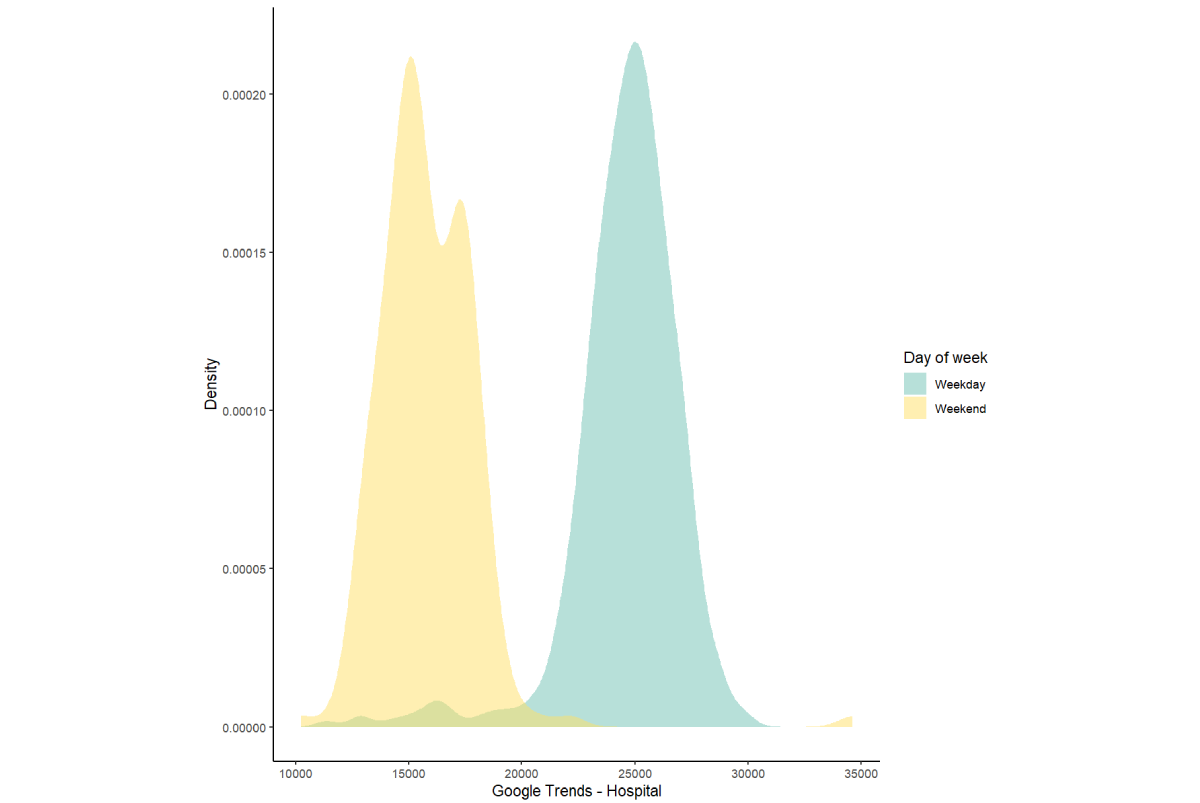
Density plot of Google Trends “hospital” data.

**Figure 3:**
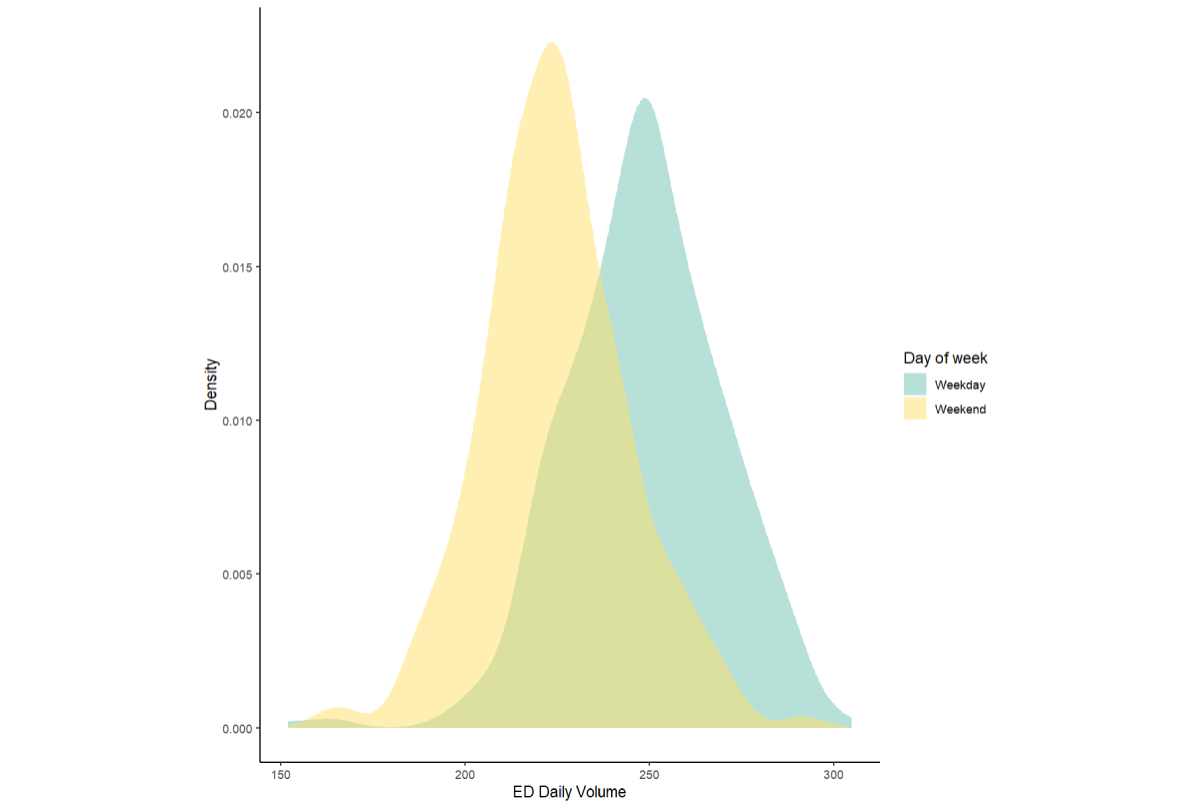
Density plot of emergency department (ED) daily volume.

The daily Google Trends relative frequency for most terms demonstrated properties of a normal distribution, with the exception of those for “shortness of breath,” “hospital,” or for all terms combined (Figure 1). The relative search frequencies for “hospital” and all terms combined exhibited a bimodal distribution; the bimodal distribution for “hospital” data was largely explained by weekday and weekend differences (Figure 2). A similar pattern was evident in emergency department daily volume (Figure 3). Two terms, “blood stool” and “blood urine,” did not yield any relative frequency data and, therefore, were excluded from subsequent analyses. When search terms occur infrequently, Google does not share these data in order to safeguard user privacy.

**Figure 4:**
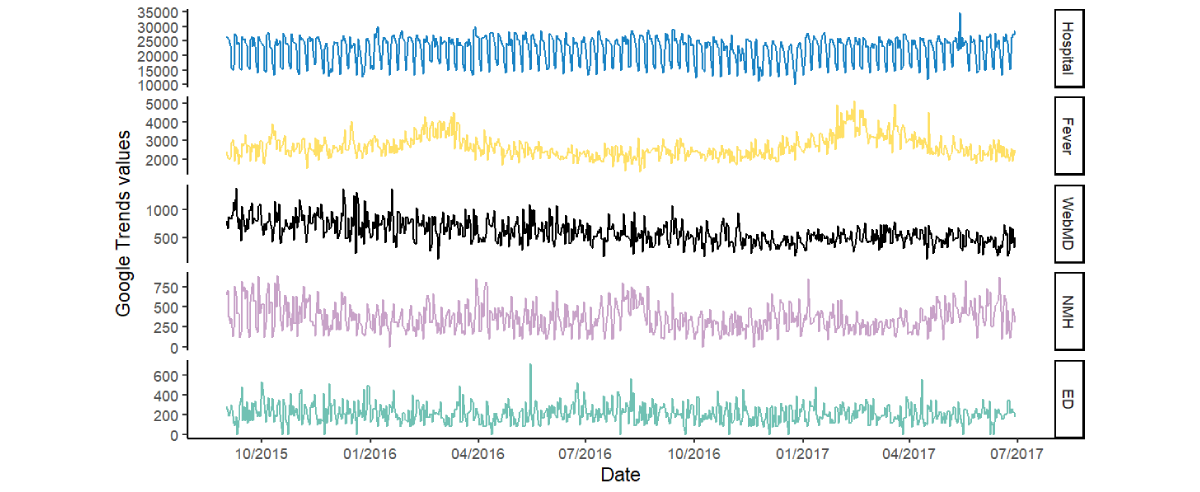
Google Trends search query data time series for the terms "hospital" (blue), "fever" (yellow), "WebMD" (black), "Northwestern Memorial Hospital" (NMH, pink), "emergency department" (ED, green).

Visual analysis of Google Trends search query data time series demonstrated 3 patterns (Figure 4): seasonal, for example, “hospital” and “fever” data exhibited weekly and annual periodicity, respectively; a declining trend, such as that for “WebMD, ” and random (ie, white noise), such as that exhibited by “Northwestern Memorial Hospital” and “emergency department.”

### Correlation Analysis

Emergency department daily volume data were moderately correlated with “hospital” (*r*=0.54, *P*<.001) and combined (*r*=0.50, *P*<.001) Google Trends search query data and were weakly correlated with “Northwestern Memorial Hospital” (*r*=0.34, *P*<.001) Google Trends search query data (Table 1).

**Table 1:** Pearson correlations between Google Trends data and emergency department daily volume.

Google Trends	None			1-day lag			1-day percentage change			3-day moving average			7-day moving average	
	*r*	*P* value	*r*		*P* value	*r*		*P* value	*r*		*P* value	*r*		*P* value
Chest pain	0.00	.98	0.01		.89	0.00		.91	0.05		.18	0.05		.25
Back pain	0.11	.005	0.16		<.001	–0.04		.31	0.05		.20	0.09		.02
Abdominal pain	–0.04	.26	0.00		.92	–0.01		.77	–0.02		.63	–0.02		.53
Stomach pain	0.00	.95	0.04		.32	–0.03		.38	0.03		.40	0.04		.30
Side pain	0.05	.17	0.01		.86	0.01		.81	0.05		.22	0.06		.16
Fever	–0.06	.10	–0.03		.48	–0.02		.56	0.03		.49	0.05		.20
Cough	–0.21	<.001	–0.18		<.001	–0.01		.89	–0.03		.44	0.01		.89
Shortness of breath	0.02	.54	0.02		.54	0.03		.44	0.03		.42	0.02		.70
Headache	–0.04	.27	0.05		.17	–0.08		.04	–0.05		.21	–0.01		.76
Numbness	0.15	<.001	0.05		.19	0.05		.17	0.11		.003	0.09		.02
Weakness	0.11	.004	0.10		.007	0.00		.95	0.09		.03	0.07		.07
Combined	0.50	<.001	–0.04		.35	0.49		<.001	0.52		<.001	0.52		<.001
WebMD	0.10	.007	0.09		.03	0.02		.53	0.11		.006	0.09		.02
Emergency department	0.02	.61	0.01		.81	–0.05		.19	–0.04		.30	–0.03		.43
Hospital	0.54	<.001	–0.04		.27	0.51		<.001	0.53		<.001	0.53		<.001
NMH^a^	0.34	<.001	0.02		.53	0.30		<.001	0.34		<.001	0.30		<.001
NMH share	0.12	.002	0.04		.25	0.07		.06	0.09		.02	0.08		.048

^a^NMH: Northwestern Memorial Hospital.

The transformations of Google Trends search query data to explore lagging or leading indicators did not uncover hidden correlations with emergency department daily volume.

### Predictive Model Development

The application of recursive feature elimination to the candidate set of traditional variables resulted in an optimal model that utilized the Day-of-week predictor; with Sunday as the reference level, this traditional model is characterized by decreasing magnitudes of regression coefficients as the week progresses from Monday to Sunday (Table 2).

**Table 2:** Regression coefficients for traditional and Google Trend data–augmented linear regression models for total emergency department daily volume.

Variable		Traditional, beta (95% CI)	Google Trends, beta (95% CI)
Intercept		223 (219, 227)	242 (240, 243)
**Day of week**			
	Sunday	Reference	Reference
	Monday	40 (35, 46)	—^a^
	Tuesday	27 (22, 33)	—
	Wednesday	18 (12, 23)	—
	Thursday	17 (11, 23)	—
	Friday	25 (19, 30)	—
	Saturday	3.1 (–2.5, 8.7)	—
Northwestern Memorial Hospital		—	3.5 (1.8, 5.1)
Hospital 1-day percentage change		—	5.5 (–4.0, 15)
Hospital 3-day moving average		—	17 (4.4, 29)
Combined 1-day percentage change		—	1.6 (–8.3, 11)
Combined 3-day moving average		—	–11 (–24, 1.7)

^a^The predictor was not included in the model.

For the model augmented with Google Trends predictor variables, the application of recursive feature elimination yielded a model that excluded *Day of week* and contained the *Northwestern Memorial Hospital*, *Hospital 1-day percentage change*, *Hospital 3-day moving average*, and *Combined 1-day percentage change* Google Trends predictors (Table 2). When comparing the traditional and Google Trends data–augmented models, the *y*-axis intercepts were largely similar, although the *y*-axis intercept of the Google Trends data–augmented model was identical to the median emergency department daily volume of this data set.

For emergency department daily volume predictions by Emergency Severity Index level, recursive feature elimination produced models that utilized *Combined 3-day moving average* for every level and *Hospital 3-day moving average* for level 2 (Table 3).

**Table 3:** Regression coefficients for traditional and Google Trends data–augmented linear regression models for daily volume by Emergency Severity Index.

Model and variable					ESI^a^ 1, beta (95% CI)			ESI 2, beta (95% CI)			ESI 3, beta (95% CI)			ESI 4, beta (95% CI)			ESI 5, beta (95% CI)
**Traditional model**																	
	Intercept			5.9 (5.3, 6.5)			83 (80, 85)			93 (91, 95)			36 (34, 37)			3.3 (2.9, 3.7)	
	**Day of week**																
		Sunday	Reference			Reference			Reference			Reference			Reference		
		Monday	1.0 (0.20, 1.9)			24 (21, 28)			12 (8.4, 15)			2.2 (–0.11, 4.5)			0.44 (–0.16, 1.0)		
		Tuesday	–0.08 (–0.92, 0.75)			19 (16, 23)			6.9 (3.6, 10)			–0.40 (–2.7, 1.9)			0.30 (–0.30, 0.90)		
		Wednesday	0.46 (–0.37, 1.3)			16 (12, 20)			2.4 (–0.90, 5.7)			–1.1 (–3.4, 1.3)			0.21 (–0.39, 0.81)		
		Thursday	0.06 (–0.78, 0.90)			15 (11, 19)			2.8 (–0.53, 6.1)			–1.2 (–3.5, 1.1)			0.38 (–0.22, 1.0)		
		Friday	0.29 (–0.55, 1.1)			19 (15, 23)			4.7 (1.4, 8.0)			0.30 (–2.0, 2.6)			0.27 (–0.33, 0.88)		
		Saturday	–0.30 (–1.1, 0.54)			1.8 (–1.8, 5.4)			–0.62 (–3.9, 2.7)			1.7 (–.059, 4.1)			–0.24 (–0.85, 0.36)		
**Augmented model**																	
	Intercept			6.1 (5.9, 6.3)			96 (95, 97)			97 (96, 98)			36 (35, 37)			3.5 (3.3, 3.6)	
	Combined 3-day moving average			0.30 (0.08, 0.53)			–7.0 (–12, –1.6)			3.8 (2.9, 4.7)			0.33 (–0.30, 1.0)			0.18 (0.01, 0.34)	
	Hospital 3-day moving average			—^b^			15 (9.2, 20)			—			—			—	

^a^ESI: Emergency Severity Index.

^b^The predictor was not included in the model.

### Model Performance

We observed that Google Trends data–augmented models generally had superior prediction performance compared to the traditional model, when based on MAE; however, these improvements were minimal (Table 4).

**Table 4:** Predictive performance of total and Emergency Severity Index daily volume for traditional and Google Trends data–augmented models.

Model			Traditional model, mean absolute error^a^		Augmented model, mean absolute error		Change (%)
All visits			15.69		15.21		–3.1
**Emergency Severity Index**							
	1	2.52		2.41		–4.7	
	2	10.37		10.66		2.8	
	3	9.55		9.12		–4.5	
	4	6.93		6.85		–1.2	
	5	1.80		1.74		–3.6	

^a^In units of patients/day.

The MAPE of the traditional model was 6.67%, and the MAPE of the Google Trends data–augmented model was 6.42%; MAPE was not calculated for models by Emergency Severity Index since they contained records with 0 daily volume, which would produce an undefined result (ie, the denominator would have been 0 in these instances).

## Discussion

### Principal Results

The goal of this study was to evaluate the potential of Google Trends search query data of healthcare facilities and chief complaints to improve the prediction performance of ED daily volume of a large-volume, tertiary-care, adult hospital. The use of Google Trends search query data to forecast emergency department daily volume resulted in a marginal improvement (MAE 3.1%) in prediction performance compared to that of a traditional prediction model. This is a small but notable improvement; when one considers that the original Google Flu Trends model included data from a set of 45 unique search queries, the ability of this study’s narrow list of Google Trends terms to produce forecast results similar to traditional models highlights the potential for this alternative real-time data source to be honed further with more advanced models and a more expansive set of Google Trends term candidates [4]. Alternatively, one may conclude that the prediction capabilities of traditional and Google Trends data–augmented models were roughly similar. The finding that Google Trends search query data alone reproduced similar predictions to those made with conventional calendar day variables demonstrates the utility of Google Trends search query data in signaling health information–seeking behavior from prospective emergency department patients.

A notable strength of this study was the use of daily emergency department encounter data. A common obstacle that infodemiology researchers face is the lack of accessible, high-frequency, and recent hospital data, which constrains their ability to leverage the real-time and high-volume attributes of Google Trends and other social media data sources (ie, big data). As more and more collaborations leverage health care organization databases for service operations data, researchers will accelerate the development of nowcasting services that have the potential to inform and optimize service operations decisions. For example, a robust nowcasting service for emergency department daily volumes could provide hospital administrators with advanced notice of impending emergency department overcrowding and trigger the coordination of earlier mitigating responses throughout the hospital.

Unexpectedly, model coefficients for the *Combined 3-day moving average* variable were negative in the Google Trends data–augmented models of total volume (β=–11.0, 95% CI –24 to 1.7) and Emergency Severity Index 2 (β=–7.0, 95% CI –12 to –1.6). Negative coefficients may reflect that sicker patients present rapidly to emergency departments and do not have time to contemplate their illness and search the internet for information. Although, this negative coefficient result was not found in the Google Trends data–augmented Emergency Severity Index 1 model, we suspect this could be due to the small proportion of Emergency Severity Index 1 encounters that were available in this data set (4043/159,769, 2.5%). Analysis of a data set with more Emergency Severity Index 1 encounters could show results consistent with other Emergency Severity Index levels. Given that low-acuity encounters (Emergency Severity Index 3, 4, and 5) were the majority, with approximately 60% (95,861/159,769), the implication that individuals with high-acuity cases may not consult the internet prior to arriving at the emergency department would not have applied to a majority of emergency department encounters at this study site. Alternatively, these counterintuitive results of a negative coefficient value for the *Combined 3-day moving average* variable may be explained by the proximity of coefficients’ 95% confidence intervals to 0; nonetheless, it is important to present these model outputs to highlight the unbiased results from a systematic approach to model generation. Altogether, these findings of illogical regression coefficients remind us of the need exercise caution with data mining exercises and predictive models that emphasize error metrics while overlooking meaningful causal relationships.

### Comparison With Prior Work

The traditional model using a day-of-week predictor in a prior study [18] that explored forecasting daily volume at an academic children’s hospital emergency department in Boston had a larger error (MAPE 10.99%) and their Google Trends search query data–based model had a smaller improvement (MAPE 1.67%) than those found in our study (traditional day-of-week model: MAPE 6.67%; improvement: MAPE 3.1%). Although the reason for the differences in MAPE for models that employed day-of week predictors is not obvious, we hypothesize that the differences in the impact of Google Trends search query data could be due to a greater utilization of the internet to understand symptoms of an acute illness among adults compared to pediatric patients and their adult guardians. There may be a population subset whose health activity is better measured by internet and social media activity data such as in the case of suicide surveillance among 25- to 34-year-old adults in the United Kingdom [26].

### Limitations

There are several limitations that are important to consider. We only utilized the emergency department daily volume from a single hospital in Chicago, whereas the Google Trends search query data pertained to the entire metropolitan area; we may have failed to identify more meaningful predictive relationships between Google Trends search query data and emergency department daily volume since we did not include the metropolitan-wide emergency department daily volume data, nor could we identify Google Trends search queries that occurred within our study site’s geographic service area.

Moreover, we only analyzed Google Trends search query data in English, which limits our ability to extrapolate these results to regions of the country where there may be greater segments of the population that use search engines in non-English languages.

Similar limitations exist in regions of the country that face barriers to internet access, such as rural areas, although a recent survey [27] found that the gap in home broadband internet between rural and nonrural homes has decreased from 16% to 7% and overall smartphone ownership has increased from 81% to 85% between 2019 to 2021; in addition, 72% of nonbroadband users reported the ability of smartphones to accomplish all desired internet tasks [27]. Therefore, as market penetration of home broadband and smartphone ownership increases, limitations due to barriers to the internet may become less prominent.

In addition, we did not attempt to predict emergency department daily volume by type of chief complaint (eg, cardiac, respiratory, neurologic). Given the difference in scale of the Google Trends search query data across types of chief complaints, future work should focus on predicting daily volumes of categories of chief complaints using an expanded set of symptom-specific Google Trends search query data.

Lastly, we only leveraged a single source of internet data, which may have only provided a glimpse into health information–seeking behaviors from prospective emergency department patients. Other data sources, such as news media and social media platforms could be incorporated [28,29]. While more resources would be required to leverage additional data sources for more complex and potentially more accurate prediction models of emergency department daily volumes, the ability for health care systems to anticipate increased demand for emergency department services would be valuable in terms of reduced health care expenses and improved patient experiences. For instance, the potential for health care systems to identify when and where low-acuity emergency department encounters may occur could guide the strategic expansion of clinic appointment availability and required advertisements to divert potential emergency department patients into less costly and more convenient venues of care.

It is worth discussing the ongoing debate regarding the ability of infodemiology data such as Google Trends search queries to reliably supplement or entirely replace traditional epidemiological data. While Google Trends search query data offers an enticing value proposition in providing insights into a population’s internet health information–seeking behaviors in a cost-efficient manner compared with traditional epidemiology data-gathering processes, it is important to remain critical of this emerging source of population health data. In some instances, Google Trends search query data reasonably mirror traditional epidemiology data. For example, tobacco use search query data were well correlated with findings from Youth Risk Behaviors Surveillance System and National Survey on Drug Use and Health data in the United States [30], and Google Trends search query data for chest pain were found to be strongly correlated with hospital admission data for coronary heart disease from the US Centers for Disease Control and Prevention Atlas of Heart and Stroke Statistics [31]. More recently, it was also demonstrated that Google Trends COVID-19 symptom search query data were significantly correlated with new cases and deaths from this disease [32,33]. However, potential confounders such as media influence have been found to effect Google Trends data. For instance, correlations between Google Trends search query data for anosmia and ageusia and COVID-19 cases were inconsistent early in the COVID-19 pandemic, and Google Trends search query volumes showed a marked increase following the beginning of the media’s coverage of these two prominent symptoms of COVID-19 [34]. In addition, COVID-19 Google Trends search query data from Europe were poorly correlated with COVID-19 epidemiological measures and were well correlated with the occurrence of pandemic-related press releases from the World Health Organization [35]. Overall, the ability to use Google Trends search query data for epidemiologic purposes remains an active area of inquiry, and these types of data must be used cautiously for such purposes.

### Conclusion

Emergency department daily volume prediction models augmented with Google Trends search query data performed similarly to baseline models utilizing traditional variables; error metrics demonstrated modest improvements in model accuracy for overall volume and nearly all Emergency Severity Index volumes. Our results suggest that even greater improvements in emergency department daily volume predictions can be attained with a more comprehensive set of Google Trends search query terms or the addition of complementary internet data sources such as social media.

The potential for these types of prediction models to leverage near real-time information to capture health information–seeking behavior preceding emergency department encounters and to be used as a tool for health care system administrators to better anticipate patient demands and optimize resource allocation warrants further investigation.

## Abbreviations

MAE: mean absolute error
MAPE: mean absolute percentage error
